# Non-denatured yak type I collagen accelerates sunburned skin healing by stimulating and replenishing dermal collagen

**DOI:** 10.1016/j.btre.2022.e00778

**Published:** 2022-12-17

**Authors:** Caihong Fu, Shuangni Shi, Jing Tian, Hong Gu, Linyan Yao, Jianxi Xiao

**Affiliations:** a State Key Laboratory of Applied Organic Chemistry, College of Chemistry and Chemical Engineering, Lanzhou University, Lanzhou, Gansu, 730000, China; b Gansu Engineering Research Center of Medical Collagen, China; c School of Basic Medical Sciences, Lanzhou University, Lanzhou, Gansu, 730000, China

**Keywords:** Collagen, Yak, Acute sunburn, Sunburn treatment, Wound healing

## Abstract

Sunburn is one of the most common skin lesions caused by excessive UV exposure, and its incidence is highly correlated with the risks of skin cancer. A variety of drugs including corticosteroids and NSAIDs have been developed to treat acute sunburn, however, they have raised severe concerns such as poor healing efficacy and long recovery time. We have for the first time extracted non-denatured type I collagen from yak hide, which displays a canonical triple helical structure with melting temperature of 42.7 °C. The highly pure yak collagen type I (YCI) self-assembles to form well-ordered nanofibers with periodic d-bands. YCI is highly biocompatible, and it significantly promotes the proliferation and adhesion of HFF-1 cells. The sunburn healing effects of YCI has been investigated using acute skin injury mouse model. Histological analysis shows that 4 days’ treatment of YCI has resulted in the recovery of sunburned mice skin to a healthy state, indicated by pronounced acceleration of epithelization and collagen deposition. The collagen volume fraction as well as the hydroxyproline (Hyp) content of YCI-treated sunburned skin have been found to be greatly increased, confirming the enhanced regeneration of collagen. YCI creams and dressings have also shown superior healing capacity of sunburn by remarkably shortening the recovery time. Notably, the denatured collagen-targeted staining results indicated a large quantity of denatured collagen in sunburned mice, which became substantially reduced after the YCI treatment. FITC-labeled YCI has been further found to penetrate into the dermis of sunburned mice. The highly biocompatible and bioactive non-denatured YCI provides an improved treatment of sunburn, indicating very promising applications of YCI in cosmetics and dermatology.

## Highlights

• Non-denatured type I collagen has been for the first time extracted from yak hide.
• The highly pure yak collagen type I (YCI) significantly promotes the proliferation and adhesion of HFF-1 cells.
• Acute skin injury mouse model studies have demonstrated the superior efficacy of YCI to treat sunburn.
• YCI accelerates sunburned skin healing by stimulating and replenishing dermal collagen.
• The highly biocompatible and bioactive YCI provides an improved treatment of sunburn, indicating very promising applications of YCI in cosmetics and dermatology.

## 1 Introduction

Sunburn is one of the most common skin diseases caused by excessive UV exposure [1]. Skin is a complex and critical organ mainly composed of epidermis and dermis, which acts as a barrier between the interior of the body and the external environment, protecting the body from external damages [2]. Most of the ultraviolet rays in sunlight are UVA with a wavelength of 320–400 nm, and about 50% of the UVA can penetrate into the epidermis [3]. Sunlight also contains a small amount of UVB with a wavelength of 280–320 nm, most of which can be absorbed by the epidermis [4]. Severe UV radiation was found as the main cause of many acute conditions of the skin, such as erythema, inflammation, papules, blisters, flaking, and hyperpigmentation [5]. The incidence of sunburn has been reported to increase year by year, and an investigation has found that more than one-third of the U.S populations have endured sunburn at least once a year [6]. The increased prevalence of sunburn has been considered to significantly exacerbate the risk of skin cancer [7].

A variety of topical medications have been developed for sunburn treatments, including corticosteroids, non-steroidal anti-inflammatory drugs (NSAIDs), antioxidants, anesthetics and antihistamines [8]. Topical corticosteroids have been commonly used to cure sunburn, but its efficacy remains controversial [9]. It has been reported that the use of topical corticosteroids failed to reduce acute sunburn reactions [10]. NSAIDs have been found to be only beneficial in early erythema [11]. Polyphenols derived from plants such as green tea, grape seeds, and honey bush are excellent antioxidants, and they have been widely used to prevent UV damage, while their curative effects of sunburn are barely understood [12]. Anesthetics can be used to relieve sunburn symptoms, but it may result in severe risks of allergic contact dermatitis using topical anesthetics containing benzocaine [13]. Antihistamines have also been used to treat sunburn, but their underlying mechanism remains obscure [14]. It has been found that most drugs display poor performance in shortening the recovery time of sunburn [15]. Better medicines are urgently needed to be developed for the treatments of sunburn.

Collagen is the major component of the dermis, while the damage of collagen has been widely known as a key feature of acute sunburn. It has been reported that the excessive exposure to UV radiations resulted in severe injury associated with collagen, including decreased procollagen mRNA expression [16], collagen polypeptide chain breakage and cross-linking of collagen fibers [17]. At the initial stage of UV irradiation, the triple helix of collagen was stabilized by UV-induced cross-linking. As the irradiation continued, the triple helix became disrupted and denatured, and the ultimate tensile strength and elongation of collagen tendons got decreased with increasing UV exposure time [18]. Serious lesion also occurred in collagen fibril d-bands, while the height difference of overlapping and gap regions was reduced from 3.7 nm to 0.8 nm, and the fibril diameter was decreased by an average of 8–10% [19]. Furthermore, the enhanced production of MMP family members (MMP-1, MMP-2, MMP-3 and MMP-9) has been shown to accelerate the degradation of collagen [20].

Animal collagen has been widely used in the treatment of skin wounds due to its attractive properties such as high biocompatibility and bioactivity [21,22]. Collagen has been found to play a critical role in almost the whole process of wound repair, including triggering platelet activation in primary hemostasis, activating the expression of coagulation factors VIII, IX, XI and XII in secondary hemostasis, and promoting cell growth and proliferation during tissue remodeling [23]. Recently, collagen extracted from different sources have displayed pronouncedly different properties such as the amino acid composition, the alignment of collagen fibers, the thermal transition temperature and the enzyme sensitivity [24]. The physicochemical features of collagen have also been reported to strongly depend on the extraction methods [25,26]. Bovine skin and tendons are major industrial sources of collagen, which has broad applications in tissue engineering and regenerative medicine [27]. Fish collagen has been widely used as food additives [28,29], and it has been found that fish collagen is much less stable than bovine collagen [30,31].

Yak is a unique cattle species growing in the plateau with excessive UV exposure [32]. Compared with low-altitude cattle, yak has been found to be enriched in protein domains involved in sensing the extracellular environment and hypoxic stress after thousands of years of adaption [33]. Herein, we have for the first time prepared non-denatured type I collagen from yak hide. Yak collagen type I (YCI) displays perfect triple-helix structure with a melting temperature of 42.7 °C, which self-assembles to form fibers with the characteristic d-banding. YCI is highly biocompatible, and it remarkably promotes the proliferation and adhesion of human skin fibroblasts. Notably, YCI, YCI creams and dressings have all shown excellent healing efficacy of sunburned skin by accelerating the recovery process. The bioactive, non-denatured yak collagen provides a potent treatment of sunburn, which have broad applications in cosmetics and dermatology.

## 2 Materials and methods

### 2.1 Preparation of type I collagen from yak hide

The hide of yak was washed and pulverized into small pieces after the foreign matters were removed. The small pieces were treated with 1% chlorhexidine and 5% hydrogen peroxide, and then washed with water. 10% n-butanol was used for degreasing. The precipitates were collected and washed with water. The harvested precipitates were decalcified with 0.5 M hydrochloric acid and then washed with water. The collected precipitates were soaked in 0.5 M sodium hydroxide and stirred at 10 °C for 2 hrs. The precipitates were obtained and then treated with 0.5 M acetic acid solution containing 1 g/L pepsin. pH was adjusted to neutral to inactivate the enzyme. The extracted yak collagen was purified by dialysis, and freeze-dried. The temperature of the whole process was maintained lower than 25 °C. Pure yak collagen type I (YCI) was stored at −20 °C.

### 2.2 Electron microscopy

SEM images of yak collagen type I (YCI) were recorded by using a Hitachi S-4800 scanning electron microscope (Hitachi Limited, Japan) with an operating voltage of 5.0 kV. YCI with a concentration of 1 mg/mL was freeze-dried and sputter-coated with AuPd for 2 min prior to imaging. TEM images were obtained on a Talos F200S transmission electron microscope (FEI, America). YCI with a concentration of 1.5 mg/mL was prepared in PBS buffer (20 mM, pH 7.4). Images were recorded at 200 kV.

### 2.3 Circular dichroism spectroscopy

Circular dichroism (CD) spectra were acquired on a CD spectrometer (JASCO J1500) equipped with a temperature controller. The YCI solutions with a concentration of 1.0 mg/mL were prepared in phosphate buffer (20 mM, pH 7.0). The samples were equilibrated for at least 24 hrs at 4 °C prior to the CD measurements. Cells with a path length of 1 mm were used. Wavelength scans were carried out from 190 to 260 nm with a 1.0 nm increment per step and a 1.0 s averaging time. Thermal unfolding curves were measured by monitoring the amplitude of the characteristic CD band at 225 nm, while the temperature was increased by 1 °C/min from 4 °C to 20 °C, 0.3 °C/min from 20 °C to 50 °C, and 1 °C/min from 50 °C to 80 °C with an equilibration time of 1 min at each temperature. The melting temperature (Tm) was estimated from the first derivative of the thermal unfolding curves.

### 2.4 Cytotoxicity

HeLa cells were grown to a cell density of 1 × 10^5^ cells/mL in complete cell culture medium (1% FBS, 2% Penicillin–Streptomycin, DMEM medium) in a humidified atmosphere of 5% CO_2_ at 37 °C. The cells were plated at a density of 5000 cells per well in a 96-well culture plates, and incubated for 24 hrs to allow for attachment. The cells were treated with different concentrations of YCI (0.01, 0.05, 0.1, 0.5, 1 mg/mL) for 24 hrs. The cells cultured in DMEM in the absence of YCI was used as control group. 10 μL of CCK-8 reagent was added into each well, and incubated for 4 hrs at 37 °C in darkness. The optical density at a wavelength of 450 nm was measured using a Tecan Infinite F200/M200 multifunction microplate reader (Tecan, Männedorf, Switzerland). Cell viability was determined as the mean absorption value of the YCI-treated group divided by the mean absorption value of the control group.

### 2.5 Cell proliferation

Human skin fibroblasts (HFF-1) cells were grown in complete cell culture medium (1% FBS, 2% Penicillin–Streptomycin, DMEM medium). The cells were plated at a density of 5000 cells per well in a 96-well culture plates, and incubated in a humidified atmosphere of 5% CO_2_ at 37 °C for 24 hrs. 0.1 mg/mL YCI in DMEM medium was added to the wells. The cells cultured in DMEM in the absence of YCI was used as control group. The cells were cultured in a humidified atmosphere of 5% CO_2_ at 37 °C for 24 hrs, 48 hrs, 72 hrs and 96 hrs, respectively. 10 μL of CCK-8 reagent was added into each well, and incubated for 4 hrs at 37 °C in darkness. The optical density at a wavelength of 450 nm was measured using a Tecan Infinite F200/M200 multifunction microplate reader (Tecan, Männedorf, Switzerland). Cell viability was calculated as the mean absorption value of the YCI-treated group divided by the mean absorption value of the control group.

### 2.6 Cell adhesion

0.1 mg/mL YCI solution was added to the wells in a 24-well cell culture plate without TC treatment. Heat-denatured BSA was added in other wells of the plate as control. Human skin fibroblasts (HFF-1) cells were grown to a cell density of 3 × 10^5^ cells/mL in high-glucose DMEM medium in a humidified atmosphere of 5% CO_2_ at 37 °C. The plate was air-dried and 500 μL of cell suspension was added to each well. The cells were then cultured in a humidified atmosphere of 5% CO_2_ at 37 °C for 5 hrs. The images were acquired using a Leica fluorescence microscope (Leica Microsystems Inc., Wetzlar, Germany).

### 2.7 Preparation of YCI cream and dressing

YCI cream was made of the following components: 15% YCI, 10% Butylene Glycerin, 8% Glycerin, 7% Betaine, 6% Caprylic/Capric Triglyceride, 6% Polysiloxane, 3% wax stearyl glucoside, 1% marigold flower extract, 1% glyceryl stearic acid, 1% hyaluronic acid, 0.2% phenoxyethanol and 0.1% centella asiatica extract. The phase A mixture (caprylic/capric triglyceride, cyclohexane, dimethicone, glyceryl stearic acid, cetearyl glucoside) and the phase B mixture (water, glycerol, betaine, sodium hyaluronate) were stirred and heated to 90 °C, respectively. The two phases were then mixed, emulsified and homogenized for 5 min. After the two phase mixture was cooled down to 45 °C, the phase C materials (butanediol, Centella asiatica extract, mother chrysanthemum extract, phenoxyethanol) were added and homogenized for 5 min. When the mixture was cooled down to 35 °C, YCI was added. Finally, the mixture was cooled down to 30 °C, and filtered to obtain the YCI cream.

The formula of YCI dressing was as follows: 10% YCI, 10% butanediol, 8% erythritol, 1% shea extract, 5% glycerin, 3% niacinamide, 2% transparent Acid, 0.5% bisabolol and 0.14% methylparaben. The phase A mixture (water, glycerol, erythritol, niacinamide, hyaluronic acid) was heated to 85 °C and stirred until complete dissolution. The phase A mixture was then homogenized for 3 min and incubated for 15 min. When the mixture was cooled to 70 °C, butanediol solution was added. When the temperature was decreased to 40 °C and 35 °C, the phase C mixture (methylparaben, bisabolol) and the phase D mixture (shea extract, YCI) were added, respectively. Finally, the mixture was kept stirring to reach transparent solution, and the YCI dressing was achieved after filtering.

### 2.8 Animal experiments

All animal experiments were performed with protocols approved by the ethics committee of Lanzhou University No.1 Hospital. Adult female mice between 6 and 8 weeks of age were purchased from Lanzhou Veterinary Research Institute (Lanzhou, China). All animals were fed with standard laboratory diets. 36 mice were used to create acute UV-injured skin model. The back of all the mice was shaved with a hair clipper, and then depilated with a depilatory cream. The depilation area was about 2 cm x 4 cm. 36 depilated mice were randomly divided into 6 groups (Normal, Untreated, YCI, YCI cream, YCI dressing, FITC-YCI), and each group had 6 mice. Mice models of UV-mediated acute skin injury was generated by UV irradiation as described previously [34,35]. Briefly, back skin depilated of hair was exposed to UVA and UVB irradiation using UVA (320–440 nm) and UVB (280–320 nm) lamps (Nanjing Huaqiang Electronics Co., Ltd., China). UVA and UVB irradiation of 100 mJ/cm^2^ was applied to induce skin inflammation. Three different types of treatments (1.0 mg/mL YCI solution, YCI cream and YCI dressing) were carried out following UV exposure. The UV-irradiated mice without any treatment were used as negative control. Healthy mice without any irradiation were used as normal group for comparison. For each group, 3 mice were sacrificed on day 2 and day 4, respectively.

### 2.9 Histological analysis

Skin from euthanized mice models of UV-mediated acute skin injury were harvested. The skin tissues were fixed in 4% paraformaldehyde in 10 × 10^−3^ M PBS (pH 7.4) for 24 hrs and embeded in paraffin. The tissues were sectioned to 3.5 µm thickness on poly-lysine treated glass slides. Sections were stained with H&E and Masson's Trichrome to evaluate epidermization, inflammation, cellular infiltration, dermal thickness and collagen density/structure. The stained tissue sections were imaged on a Leica DM4000B metallurgical upright microscope (Leica Microsystems Inc., Wetzlar, Germany).

### 2.10 Quantitative analysis of malondialdehyde, collagen volume fraction and hydroxyproline

The content of Malondialdehyde (MDA) was determined according to the procedures of Malondialdehyde Assay Kit (Solarbio, Beijing, China). Briefly, the condensation of MDA with thiobarbituric acid (TBA) led to red products, with the maximum UV absorption peak at 532 nm. The MDA content (nmol/mg fresh weight) was defined as: 5 × (12.9 × (A_532_ -A_600_) - 2.58 × A_450_) / W. A_532_, A_600_ and A_450_ are the UV absorbance at 532 nm, 600 nm and 450 nm, respectively. W represents sample mass. The collagen volume fraction (CVF) was defined as the percent area fraction CVF (%) = (area of collagen fibers/area of skin tissue) × 100%, and derived from Masson trichrome stained histologic sections using Image J software.

The concentration of hydroxyproline was determined in order to evaluate the collagen content in the skin tissues of model mice following previously described procedures. Briefly, 20–30 mg of fat-removed skin tissues were minced, and incubated with 0.5 M acetic acid and 10 g/L pepsin for 12 hrs. The supernatants were collected, and treated with 6 N hydrochloric acid at 110 °C for 16 hrs to allow complete hydrolysis of collagen. pH of the solution was then adjusted to 7.0 at room temperature. Standard solutions of hydroxyproline were prepared at concentrations of 1.0–100 μg/mL. Chloramine T was added to the solution, and incubated at room temperature for 20 min. Ehrlich's reagent was then added, and incubated at 60 °C for 20 min. The mixture was cooled to room temperature and incubated for 30 min. The UV absorption at 560 nm was measured, and the concentrations of hydroxyproline in the hydrolytic samples were determined according to the standard curve. The concentrations of hydroxyproline in tissue homogenates were determined per milligram of dry tissue.

### 2.11 Targeted detection of denatured collagen

Denatured collagen in the skin tissues of model mice was detected using a specific denatured collagen-targeting fluorescent peptide probe FAM-(GPO)_8_ as previously described [36]. Briefly, the skin tissues were fixed in 4% paraformaldehyde, embeded in paraffin, and sectioned to 3.5 µm thickness. After the removal of paraffin, 10% goat serum was added to the tissue sections to block nonspecific binding. 100 μL solution of the peptide probe with a concentration of 15 μM was added onto each tissue slide, and incubated at 4 °C for 5 hrs. The tissue slides were washed using 10 mM PBS five times to eliminate unbound peptide probes. A drop of antiquenching agent was added on the tissue slide. The stained tissue sections were imaged on a Leica DM4000B metallurgical upright microscope (Leica Microsystems Inc., Wetzlar, Germany). Fluorescence intensity of each FAM-(GPO)_8_ stained skin tissue was determined by Image J software.

### 2.12 Treatment of UV-irradiated mice skin by FITC-YCI

FITC-labeled YCI (FITC-YCI) was used to investigate the penetration capability of YCI on sunburned skin. FITC-YCI was synthesized following the procedure below. 10 mL of 0.5 mol/L Na_2_CO_3_ solution was mixed with 90 mL of 0.5 mol/L NaHCO_3_ solution, and the pH of the mixture was adjusted to 9.0. 1 mg/mL YCI solution was prepared in 0.5 mol/L Na_2_CO_3—_NaHCO3 buffer with 0.15 mol/L NaCl. 10 mg Fluorescein isothiocyanate (FITC) was added to the mixture, and kept stirring at 4 °C for 24 hrs to allow complete reaction. The solution was dialyzed using a dialysis membrane with molecular weight cutoff of 2000 Da in 20 mmol/L PBS buffer (pH 7.4). No fluorescence was detected in the dialysate, indicating complete removal of remnant free FITC. The obtained FITC-YCI solution was applied to treat UV-irradiated skin of 6 mice following the same protocol as YCI. The FITC-YCI-treated mice skin section was characterized by Masson staining as described above. Fluorescent images were recorded on a fluorescence microscope (Leica Microsystems Inc., Wetzlar, Germany). The fluorescent collagen fibers area and the total area of collagen fibers was estimated by Image J software. The relative content of FITC-YCI (%) was calculated as the ratio of fluorescence area to total collagen area.

### 2.13 Statistics

For the quantitative analysis of the content of MDA and Hyp, collagen volume fraction, and fluorescence imaging results of denatured collagen and FITC-YCI, the Student's *t*-test was utilized to compare the two independent groups. Differences were considered to be statistically significant when the p value was < 0.05.

## 3 Results and discussion

### 3.1 Characterization of type I collagen from yak hide (YCI)

**Fig. 1:**
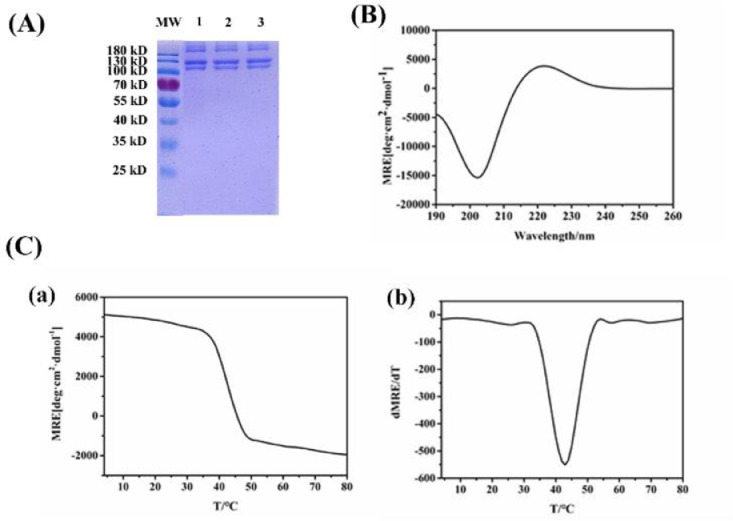
SDS-PAGE and Circular Dichroism characterization of YCI. (A) SDS-PAGE of molecular weight standard (MW) and YCI produced in three different batches; (B) CD spectrum of YCI; (C) CD thermal transition curve of YCI; (D) The first derivative of the thermal unfolding curve of YCI.

Type I collagen was extracted from yak hide by pepsin treatment, while the whole process was maintained at temperatures lower than 25 °C. SDS-PAGE was used to confirm the identity and purity of YCI (Fig. 1A). Two bands near the molecular weight of 100 kDa were observed, which corresponded to the two α chains (α1 and α2) of type I collagen. Two other bands (β) near the molecular weight of 200 kDa were assigned to the dimer species composed of two α chains, which was consistent with previous reports. The absence of any extra bands at lower molecular weight indicated high purity of YCI without degradation.

Circular dichroism (CD) was performed to determine the secondary structure of YCI, while a positive peak near 221 nm and a negative peak near 198 nm were indicative of the triple helix structure of collagen [37,38]. CD spectra of YCI showed a positive peak around 221 nm and a negative peak around 198 nm, demonstrating the formation of distinct triple helical structure (Fig. 1B) [39]. The amplitude of the CD absorption at 225 nm was monitored from 4 °C to 80 °C to obtain the thermal transition curve, while the melting temperature (Tm) was calculated from the first derivative of the thermal unfolding curves (Fig. 1C-D). The melting temperature of YCI was estimated as 42.7 °C, which was similar as that of the bovine collagen [31].

**Fig. 2:**
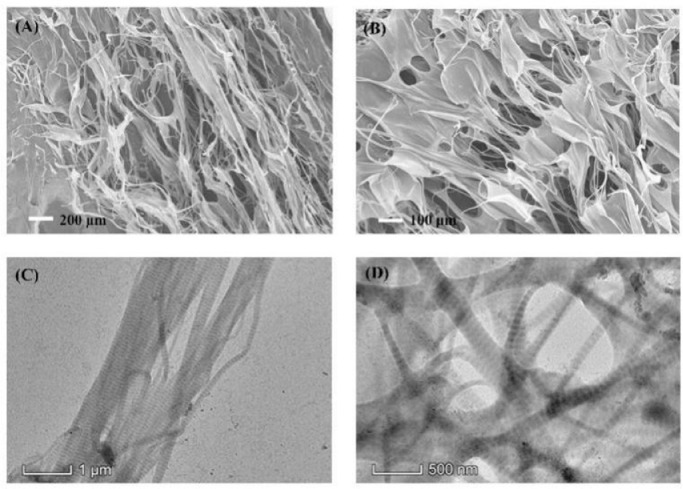
SEM (A-B) and TEM (C-D) characterization of YCI.

Scanning electron microscopy (SEM) and Transmission electron microscopy (TEM) were applied to characterize the morphology of YCI. SEM images showed that YCI formed a well-ordered three-dimensional fibrous network (Fig. 2A-B). TEM images displayed exquisite nanofibers with distinct periodic d-banding (Fig. 2C-D). The SEM and TEM results suggested that the YCI triple helices self-assembled to form hierarchical fibrous nanostructures, which were similar as collagen extracted from other sources [40,41].

### 3.2 Biocompatibility and bioactivity of YCI

**Fig. 3:**
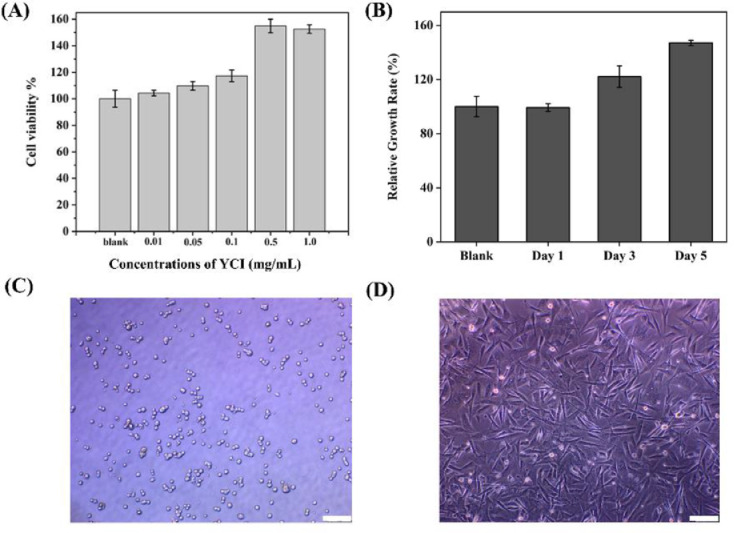
. Biocompatibility and bioactivity of YCI. (A) In-vitro cytotoxicity of YCI; (B) The relative proliferation rates of human skin fibroblasts in YCI; (C) Images of HFF-1 cells on BSA substrates; (D). Images of HFF-1 cells attached to YCI substrates. Scale bars:100 μm.

The biocompatibility of YCI was evaluated by cytotoxicity and cell proliferation experiments. The in vitro cytotoxicity of YCI was determined by examining the viability of HeLa cells using the CCK-8 assays (Fig. 3A). Compared with the blank, the viability of the cells grown in YCI solutions with different concentrations (0.01–1.0 mg/mL) was all greater than 100%, indicating that YCI was nontoxic. Furthermore, the relative proliferation rates of human skin fibroblasts (HFF-1) in 0.1 mg/mL YCI solution gradually increased to 120% on the 3rd day and 150% on the 5th day, indicating that YCI significantly promote the proliferation of HFF-1 (Fig. 3B). The cytotoxicity and cell proliferation results demonstrated the high biocompatibility of YCI.

The bioactivity of YCI was evaluated via cell-adhesion assay using HFF-1 cells. HFF-1 cells were cultured on the plate wells coated with YCI and bovine serum albumin (BSA). The HFF-1 cells attached to the BSA substrates maintained uniform spherical shape (Fig. 3C), while the HFF-1 cells on the YCI substrates showed well-spread pattern (Fig. 3D). The extensive cell spreading demonstrated that the YCI nanofibers provided a highly bioactive scaffold for HFF-1 cell adhesion.

### 3.3 The treatments of sunburned skin by YCI, YCI creams and YCI dressings

**Fig. 4:**
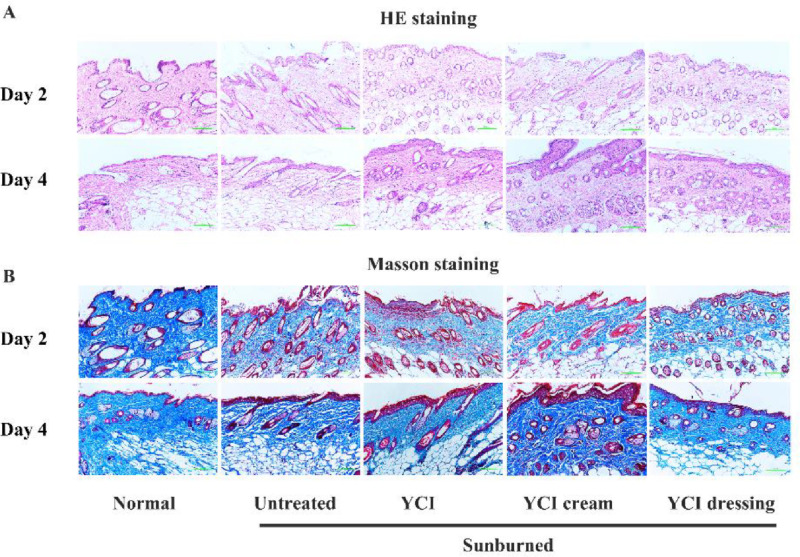
HE (A) and Masson (B) staining images of skin tissues of the normal, untreated and three treatment groups (YCI, YCI cream and YCI dressing) on day 2 and day 4. Scale bars:200 μm.

**Fig. 5:**
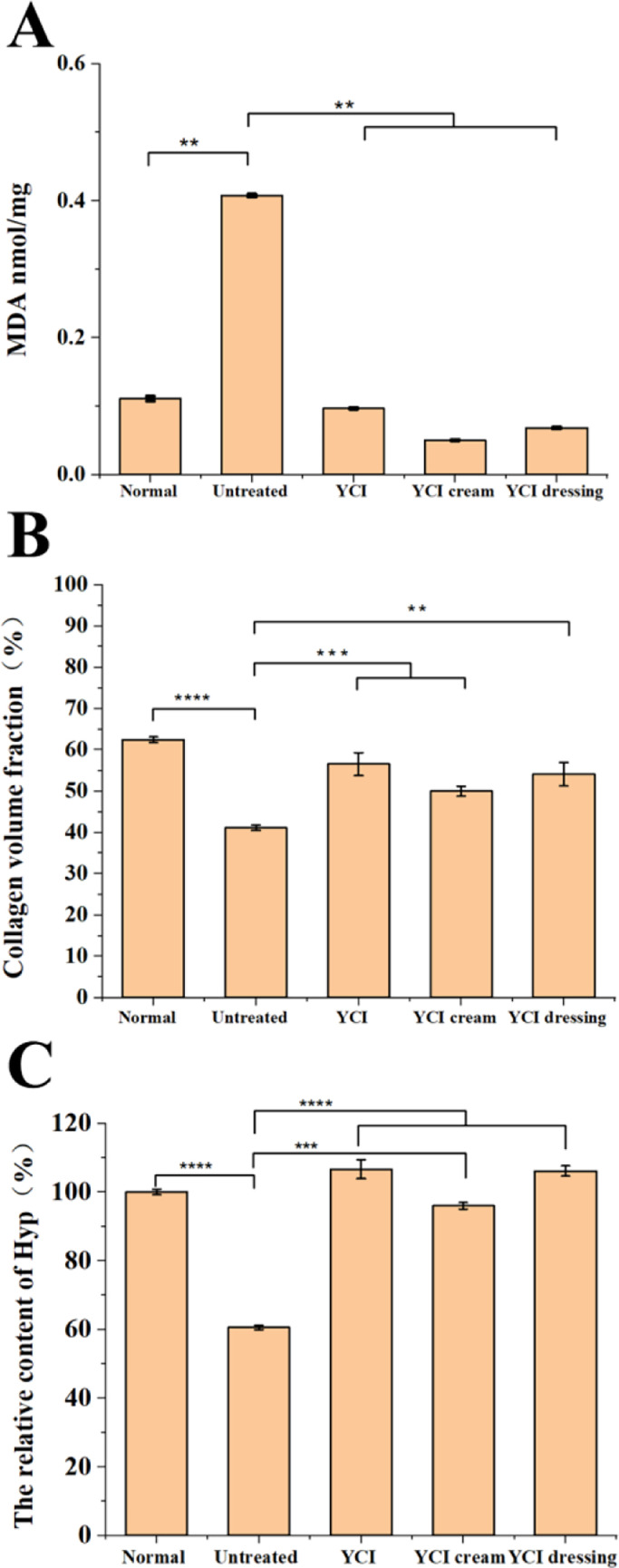
Quantitative analysis of the MDA content (A), collagen volume fraction (B) and the relative content of Hyp (C) in skin tissues of the normal, untreated and three treatment groups (YCI, YCI cream and YCI dressing). *P < 0.05, **P < 0.01, ***P < 0.001, ****P < 0.0001, ns > 0.05, *n* = 3.

UV-mediated acute skin injury mouse model was established as previously described. YCI, YCI cream and YCI dressing were applied on the severe sunburned sites, while the untreated group was used as negative control. HE and Masson staining were conducted to evaluate their healing effects (Fig. 4). For the untreated group, the HE stained sections showed epidermal damage, cellular infiltration and dermal inflammation on day 2, while the epidermis remained corroded and the dermal edema persisted on day 4, which were expected consequences by an erythemogenic dose of UV radiation (100 mJ/cm^2^) [34,35]. For the treatment group by YCI, YCI cream and YCI dressing, the HE stained sections displayed much less skin inflammation on day 2, while the epidermal layer of the sunburned sites became thicker on day 4. Furthermore, the content of MDA, a marker of oxidative stress, was significantly decreased on day 4 (Fig. 5A). Compared with the untreated group, all the three treatment groups indicated much accelerated repair of the skin wounds.

Masson staining images of the untreated group showed plenty collagen debris and short aggregates, which could result from collagen polypeptide chain breakage and degradation of collagen fibers due to severe UV irradiations (Fig. 4) [16], [17], [18]. In contrast, Masson staining images of the three treated group all showed a significant increase in the amount of well-ordered dense collagen fibers with interspersing fibroblasts, which well resembled the normal dermis. Collagen volume fraction results further demonstrated remarkable collagen deposition in the three treated groups (Fig. 5B). All the results demonstrated that YCI and its composite cream and dressing possess enhanced epithelization capability and accelerated healing efficacy for curing sunburned skin.

The hydroxyproline (Hyp) content was further measured to quantify dermal collagen in order to evaluate the collagen regeneration effects of YCI (Fig. 5C). The content of Hyp in the normal group was set as standard. Compared with the normal group, the content of Hyp was reduced to 60.59% for the untreated group, indicating a significant reduction in dermal collagen due to UV damages. After the YCI treatment, the Hyp content was increased to 106.13%, indicating an enrichment of dermal collagen. Similarly, the treatments of YCI cream and YCI dressing both led to an increase in the Hyp content (96.02% and 106.65%, respectively), demonstrating that they dramatically restored the collagen content to normal level in 4 days. These results suggested that YCI and its composite products possess the prowess to accelerate the healing of severe UV-mediated skin injury by promoting the regeneration of dermal collagen.

### 3.4 Targeted detection of denatured collagen in sunburned skin

**Fig. 6:**
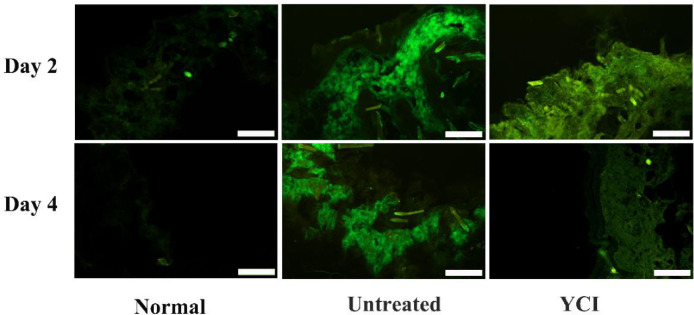
Fluorescent imaging of denatured collagen in skin sections of the normal, untreated and YCI-treated groups on day 2 and day 4. Denatured collagen was detected using a specific denatured collagen-targeting fluorescent peptide probe FAM-(GPO)_8_. Scale bars: 200 μm.

Collagen denaturation has been reported to be associated with various pathological conditions. Denatured collagen was detected to examine the health of the dermis by using a special fluorescent peptide probe FAM-(GPO)_8_, which has been demonstrated to specifically bind to denatured collagen but not intact collagen [42]. The fluorescent images of the peptide probe-stained skin sections and the calculated fluorescent integrated density of corresponding denatured collagen displayed little fluorescence for the normal group, indicating that healthy skin dermis is composed of intact triple helical collagen (Figs. 6 and 8A). In contrast, the skin tissues of the untreated group showed strong fluorescence, indicating the presence of a large amount of denatured collagen due to UV irradiation (Figs. 6 and 8A). Notably, the skin sections of the YCI group showed significantly reduced fluorescence on day 4, demonstrating a remarkable recovery of intact collagen resulting from the YCI treatment, suggesting that bioactive YCI may provide an amiable environment for inducing the regeneration of healthy triple helical collagen in UV-irradiated skin (Figs. 6 and 8A) [43], [44]. The replacement of damaged collagen by regenerated collagen may contribute to the accelerated healing of sunburned skin.

### 3.5 Evaluation of the YCI penetration into the dermis of sunburned skin

**Fig. 7:**
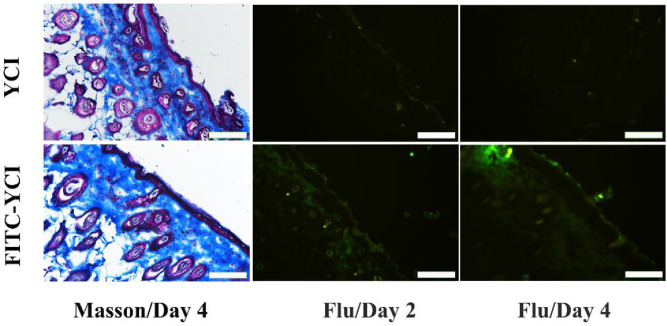
Masson staining and fluorescent images of UV-irradiated mice skin sections after the treatment by YCI and FITC-YCI. Scale bars: 200 μm.

**Fig. 8:**
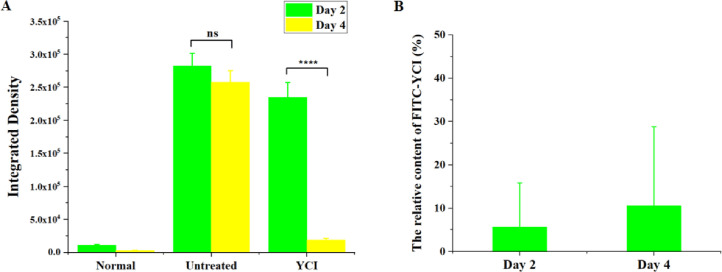
Quantitative analysis of the fluorescent integrated density of denatured collagen in the normal, untreated and YCI-treated groups on day 2 and day 4 (A), and the relative content of FITC-YCI in FITC-YCI-treated skin tissues on day 2 and day 4 (B). *P < 0.05, **P < 0.01, ***P < 0.001, ****P < 0.0001, ns > 0.05, *n* = 3.

Fluorescent dye-labeled YCI (FITC-YCI) was used to evaluate the penetration capability of YCI into the dermis of sunburned skin. FITC-YCI was synthesized, and applied to treat UV-irradiated mice skin following the same protocol as YCI (Fig. 7). The FITC-YCI and YCI treatments showed similar Masson staining results of skin sections, indicating that the dye label does not affect the curing effects of YCI on UV-irradiated skin. As a control, the fluorescent images of YCI-treated skin sections showed no fluorescence. In contrast, significant fluorescence was observed in the skin sections of FITC-YCI-treated mice, and the relative content of FITC-YCI was estimated as 5% and 10% of the total dermis collagen on day 2 and day 4, respectively (Figs. 7 and 8B), indicating the remarkable penetration of FITC-YCI into the dermis [45]. The permeation of YCI to replenish the dermal collagen may be beneficial for sunburn healing.

## 4 Conclusions

Sunburn is arguably the most prevalent skin disease due to UV-irradiation, and it has been considered as a crucial risk factor for skin cancer. Though a number of topical medications such as corticosteroids and NSAIDs have been discovered for the treatment of sunburn, their healing efficacy and underlying mechanism remains poorly understood. The development of more effective drugs to cure sunburn is still in great need. As the main component of the dermis, collagen degradation has been identified as a critical feature of acute sunburn. However, the curative effects of collagen on sunburn have so far barely investigated.

Yak is a special cattle species that have endured extensive UV exposure in the plateau. We have herein for the first time extracted highly pure non-denatured type I collagen from yak hide, which possesses perfect triple-helical structure with a melting temperature of 42.7 °C. The yak collagen type I (YCI) self-assembles to form exquisite nanofibers with distinct periodic d-banding. YCI has shown superior biocompatibility and bioactivity, which remarkably promotes the proliferation and adhesion of human skin fibroblasts.

We have further examined the sunburn healing efficacy of YCI using UV-mediated acute skin injury mice model. HE and Masson staining images of mice skin sections indicate that the untreated group showed epidermal damage, cellular infiltration and dermal inflammation, while 4 days’ treatment of YCI has resulted in the robust revitalization of sunburned mice skin evidenced by prominent acceleration of epithelization and collagen deposition. The significantly increased hydroxyproline content of YCI-treated sunburned skin has also affirmed the enhanced regeneration of collagen. Furthermore, YCI creams and dressings have shown similarly supreme sunburn healing prowess by reducing the recovery time.

The underlying mechanism of YCI treatment of sunburned skin has been investigated by denatured collagen-targeted staining and utilization of fluorescent dye-labeled YCI (FITC-YCI). A large amount of denatured collagen has been identified in UV-irradiated mice skin, which became pronouncedly reduced after the YCI treatment. In addition, the FITC-YCI studies demonstrated the possible infiltration of YCI into the dermis of sunburned mice. YCI may contribute to sunburn healing by promoting the regeneration of intact collagen as well as directly replenishing the dermal collagen. The highly biocompatible and bioactive non-denatured yak collagen and its composite products provide potent treatments of sunburn, which have broad applications in cosmetics and dermatology.

## Author statement

**Caihong Fu:** designed, conducted, analyzed, and interpreted the in vitro and in vivo data. **Shuangni Shi:** performed the cell assays. **Jing Tian:** assited the animal expriments. **Hong Gu:** assited the histological analysis. **Linyan Yao:** produced the yak collagen. **Jianxi Xiao:** directed the whole project and wrote the manucript. All authors have read and agreed to the published version of the manuscript.

## Declaration of Competing Interest

The authors declare the following financial interests/personal relationships which may be considered as potential competing interests: JIANXI XIAO reports financial support was provided by National Natural Science Foundation of China. JIANXI XIAO reports financial support was provided by Natural Science Foundation of Gansu Province.

## Data Availability

The data that has been used is confidential.
